# Human and Mouse Skeletal Muscle Stem Cells: Convergent and Divergent Mechanisms of Myogenesis

**DOI:** 10.1371/journal.pone.0090398

**Published:** 2014-02-28

**Authors:** Akshay Bareja, Jason A. Holt, Guizhen Luo, Calvin Chang, Junyu Lin, Aaron C. Hinken, Johannes M. Freudenberg, William E. Kraus, William J. Evans, Andrew N. Billin

**Affiliations:** 1 Department of Medicine, Duke University, Durham, North Carolina, United States of America; 2 Muscle Metabolism Discovery Performance Unit, Metabolic Pathways and Cardiovascular Therapeutic Area, GlaxoSmithKline, Research Triangle Park, North Carolina, United States of America; 3 Five Prime Therapeutics, Inc., South San Francisco, California, United States of America; 4 Quantitative Sciences, Computational Biology, GlaxoSmithKline, Research Triangle Park, North Carolina, United States of America; West Virginia University School of Medicine, United States of America

## Abstract

Satellite cells are the chief contributor to skeletal muscle growth and regeneration. The study of mouse satellite cells has accelerated in recent years due to technical advancements in the isolation of these cells. The study of human satellite cells has lagged and thus little is known about how the biology of mouse and human satellite cells compare. We developed a flow cytometry-based method to prospectively isolate human skeletal muscle progenitors from the satellite cell pool using positive and negative selection markers. Results show that this pool is enriched in PAX7 expressing cells that possess robust myogenic potential including the ability to give rise to *de novo* muscle *in vivo*. We compared mouse and human satellite cells in culture and identify differences in the elaboration of the myogenic genetic program and in the sensitivity of the cells to cytokine stimulation. These results indicate that not all mechanisms regulating mouse satellite cell activation are conserved in human satellite cells and that such differences may impact the clinical translation of therapeutics validated in mouse models. Thus, the findings of this study are relevant to developing therapies to combat muscle disease.

## Introduction

Satellite cells are rare, mononuclear cells located adjacent to skeletal muscle, in between the sarcolemma and the basal lamina of the surrounding extracellular matrix [1]. They are mitotically quiescent under normal conditions, and become activated in response to external stimuli like exercise or injury. Upon activation, these cells proliferate and differentiate to give rise to new muscle fibers [2]. A fraction of these cells also self-renew and are therefore considered to be *bona fide* stem cells. Satellite cells are the chief contributors to muscle regeneration in the adult [3], and are therefore thought to be of significant therapeutic value in the context of muscular dystrophies.

The satellite cell has been most extensively studied in the mouse model. In the last decade, flow cytometry-based techniques have been developed to prospectively isolate pure populations of satellite cells from their niche. These cells have been unambiguously shown to display myogenic stem cell properties, such as the ability to self-renew and give rise to differentiated muscle cells both *in vitro* and *in vivo* [4]–[6]. In contrast, considerably less research has been performed on the human satellite cell, and the methods used to isolate these cells, such as pre-plating and differential centrifugation, have many drawbacks. These drawbacks include the inability to produce a pure population of cells and, due to the length of these procedures, the isolated cells are more likely to be committed progenitor cells as opposed to genuine satellite cell derived precursors ([7]; for detailed discussion, refer to [8]).

Previous work has suggested that the cell surface marker CD34 can identify a myogenic and adipogenic precursor (CD34+, CD56+) and a myogenic but non-adipogenic precursor (CD34−, CD56+) in human skeletal muscle [9], [10]. Both cell types can contribute to myofiber formation and colonize the satellite cell niche upon transplantation into mouse muscle. The CD34−, CD56+ cells are candidate satellite cells since they have myogenic potential with no adipogenic potential, similar to their mouse counterparts [11], [12]. The work of Pisani et al [9], [10] used transplantation experiments to characterize the activity of the CD34+/CD56+ and the CD34−/CD56+ cells but provided no data comparing their properties to the mouse satellite cell. In this study we have extended the previous studies on CD34−/CD56+ myogenic cells by developing a fluorescence-activated cell sorting (FACS) protocol to isolate and obtain the cells in high purity and then determine their myogenic properties using cellular and molecular techniques. We provide evidence that the cells possess a myogenic gene expression program, have properties of satellite stem cells, and that key functions of myogenic factors are active in these cells. However, we also report key differences in the execution of the myogenic program and in the response to cytokine stimulation. This data suggests that murine and human satellite cell derived skeletal muscle precursor biology is not congruent and that such differences should be considered in the context of drug development and clinical translation.

## Materials and Methods

### Ethics Statement

The human biological samples were sourced ethically and their research use was in accord with the terms of the informed consents. Procedures were reviewed and approved by the Duke University Health System Institutional Review Board for Clinical Investigations and the GSK Global Human Biological Sample Management Governance Board. Written consent was obtained from subjects participating in the study.

For studies involving mice, all procedures performed were in compliance with the Animal Welfare Act and United States Department of Agriculture regulations and approved by the GlaxoSmithKline Institutional Animal Care and Use Committee.

### Animal Care and Models

Mice were maintained on standard laboratory chow and allowed access to food and water *ad libitum*. The following strains were used for these experiments: C57Bl6/J, B6.129S7-*Il1r1^tm1Imx^*/J, (Jackson Labs), and CB-17 SCID Beige (Charles River).

### Isolation of Human Skeletal Muscle Precursors

Subjects were healthy male and female patients (late teens to early 60′s) in need of anterior cruciate ligament (ACL) reconstruction surgery. Gracilis and sartorius muscle were stripped off the ligament with the ligament being used to replace the torn ACL. The muscle tissue that is left over (2–3 g) and usually discarded, was used for cell isolation. All samples were collected following stipulations from GSK and Duke for informed consents and anonymization of the samples. The muscle was processed for cell isolation within a few hours after excision. The muscle was distributed evenly to gentleMACS C tubes (Miltenyi Biotec) containing 15 ml of a collagenase-dispase solution (2.4 U ml^−1^ dispase, Gibco; 2 mg ml^−1^, Sigma). The muscle was incubated at 37°C in a water bath for 45 minutes and subjected to dissociation using a GentleMACS dissociator (Miltenyi Biotec) every 15 minutes. 1 ml of heat-inactivated horse serum (Invitrogen, Life Technologies) was added to each tube to halt the digestion reaction. All solutions were brought up to 40 ml by adding PBS and passed through 70 micron mesh filters (BD Bioscience), and centrifuged for 10 minutes at 1600 rpm and 4°C. The supernatant was discarded and the remaining pellet was resuspended in 2 ml of staining solution (2% heat-inactivated horse serum in HBSS), which was transferred to a 5 ml FACS tube. This solution was again centrifuged for 10 minutes at 1600 rpm and 4°C. This was repeated twice to obtain a clean pellet containing mononuclear cells. This pellet was resuspended in staining solution and stained with the following monoclonal antibodies – ICRF44 (1∶100, CD11b), WM59 (1∶100, CD31), 4H11 (1∶50, CD34), 2D1 (1∶100, CD45) (all conjugated to fluorescien isothiocyanate (FITC), eBioscience), 12G5 (1∶100, CXCR4, PE-Cy7, BD Bioscience), and NCAM16.2 (1∶50, CD56, phycoerythrin (PE), BD Bioscience). Following a 45 minute-long incubation on ice, the cell suspension was washed with staining solution and centrifuged for 10 minutes at 1600 rpm and 4°C. The resulting pellet was incubated with anti-FITC magnetic beads (1∶10, Miltenyi Biotec) for 15 minutes on ice. The cell suspension was then washed with staining solution and centrifuged for 10 minutes at 1600 rpm and 4°C. The resulting pellet was resuspended in staining solution and all the cells bound to FITC-conjugated antibodies were separated from the original suspension using the manual MACS cell separation protocol (Miltenyi Biotec), following manufacturer’s instructions. The flow-through was centrifuged for 10 minutes at 1600 rpm and 4°C. The resulting pellet was stained with calcein blue (1∶500) and propidium iodide (PI, 1∶1000). HuSMPs were identified as single, live mononuclear CD11b-CD31-CD34-CD45-CXCR4+CD56+ cells and sorted using a BD FACS ARIA II flow cytometer (Becton Dickinson). Yields varied between 50,000 to 200,000 cells per sample. These cells were either processed for downstream experiments or seeded in plates coated with collagen I (1 µg ml^−1^, BD Bioscience) and laminin (10 µg ml^−1^, BD Bioscience) in myogenic growth media (20% heat-inactivated horse serum, 1% pencillin/streptomycin and 5 ng ml^−1^ bFGF in F10). StemPro adipogenesis and osteogenesis differentiation kits were purchased from Life Technologies. All experiments were performed within two weeks of isolation and cells were not passaged. 384 well plates were typically used and seeded at 250 cells/well to 500 cells/well. All FACS data were analyzed using FlowJo.

Mouse SMPs were isolated by FACS using modifications of published protocols. Briefly, CD34 and ITGA7 were used as positive selection markers while CD31, CD45, CD11b, SCA1, and Ter119 were used as negative selection markers [13], [14].

### Gene Expression qPCR

Total RNA was isolated from fresh or cultured huSMPs following manufacturer instructions (RNAEasy Micro Plus, Qiagen). cDNA was produced using a high capacity cDNA transcription kit (Applied Biosciences). The quantitative expression of each gene was assessed using Taqman Gene Expression Assays (intron spanning to avoid amplification of genomic DNA) on an Applied Biosystems 7900HT machine or via qPCR arrays (Qiagen). Relative expression was calculated using the delta-delta C_t_ method.

### Immunocytochemistry

HuSMPs were fixed with 4% paraformaldehyde for 15 minutes, washed with PBS and permeabilized with TBS-T (0.1% Triton-X in pH 8.0 TBS) for another 15 minutes. The cells were then blocked in 2% normal goat serum (NGS) for 20 minutes. This was followed by incubation with primary antibodies for Pax7 (1∶20, DSHB), Myf5 (1∶100, Abcam), MyoD (1∶100, Dako), or myosin heavy chain (1∶500, clone A4 1025 Millipore) diluted in blocking solution for 1 hr. Alexa fluor secondary antibodies (Invitrogen) were used for detection. The cells were counterstained with Hoechst 33342 to visualize nuclei. Images were taken using fluorescent microscopes (Zeiss). Total cell number and the percentage of cells expressing a particular antigen were quantified using the Pathways 435 (BD Bioscience) and FlowJo. Myosin heavy chain staining was quantified on a GE IN Cell Analyzer.

Tissue was dissected from the mice, embedded in O.C.T. (VWR) and flash-frozen in liquid nitrogen-cooled isopentane. A cryostat (Leica) was used to make 5–8 micron-thick serial sections. Cryosections were brought up to room temperature and fixed in cold (−20°C) acetone for 5 minutes. The sections were washed with PBS and blocked in 5% NGS for 30 minutes. The sections were then incubated with human-specific primary antibody for dystrophin (MANEX1216A,1∶20, DSHB) for 1 hr, and Alexafluor secondary antibodies (1∶2000, Invitrogen) were used for detection. The sections were counterstained with Hoechst 33342 and images were taken using a fluorescent microscope (Zeiss).

### Cell Transplantation

8 week-old male CB-17 SCID Beige mice were used for the transplantation experiments. 24 hrs before transplantation, gastrocnemius and tibialis anterior muscles of both hind-limbs were injected with 50 µl of 2 µM cardiotoxin (*Naja nigricollis*, EMD Millipore). 1×10^3^ freshly-collected huSMPs in 50 µl of myogenic growth media were injected into each of the pre-injured muscles of one hind-leg and the contralateral muscles injected with growth media only. After 4 weeks the mice were sacrificed and the muscles were collected for histological assessment.

### Knockdown of Myogenic Genes

ON-TARGET *plus* siRNA pools (Dharmacon) were used to suppress the expression of human *PAX7*, *MYOD1*, and *MYF5*, in huSMPs. Transfection was performed a day after seeding using the Lipofectamine RNAiMAX reagent (Invitrogen) following manufacturer’s instructions. Cells were harvested three days later for qPCR analysis. A non-targeting siRNA pool was used as a negative control.

### Statistical Analysis

Data were analyzed using unpaired two-tailed *t*-tests. Only *p*-values <0.05 were deemed significant. All data are presented as mean +/− SEM.

## Results

### FACS Isolation of Human Skeletal Muscle Precursors

**Figure 1:**
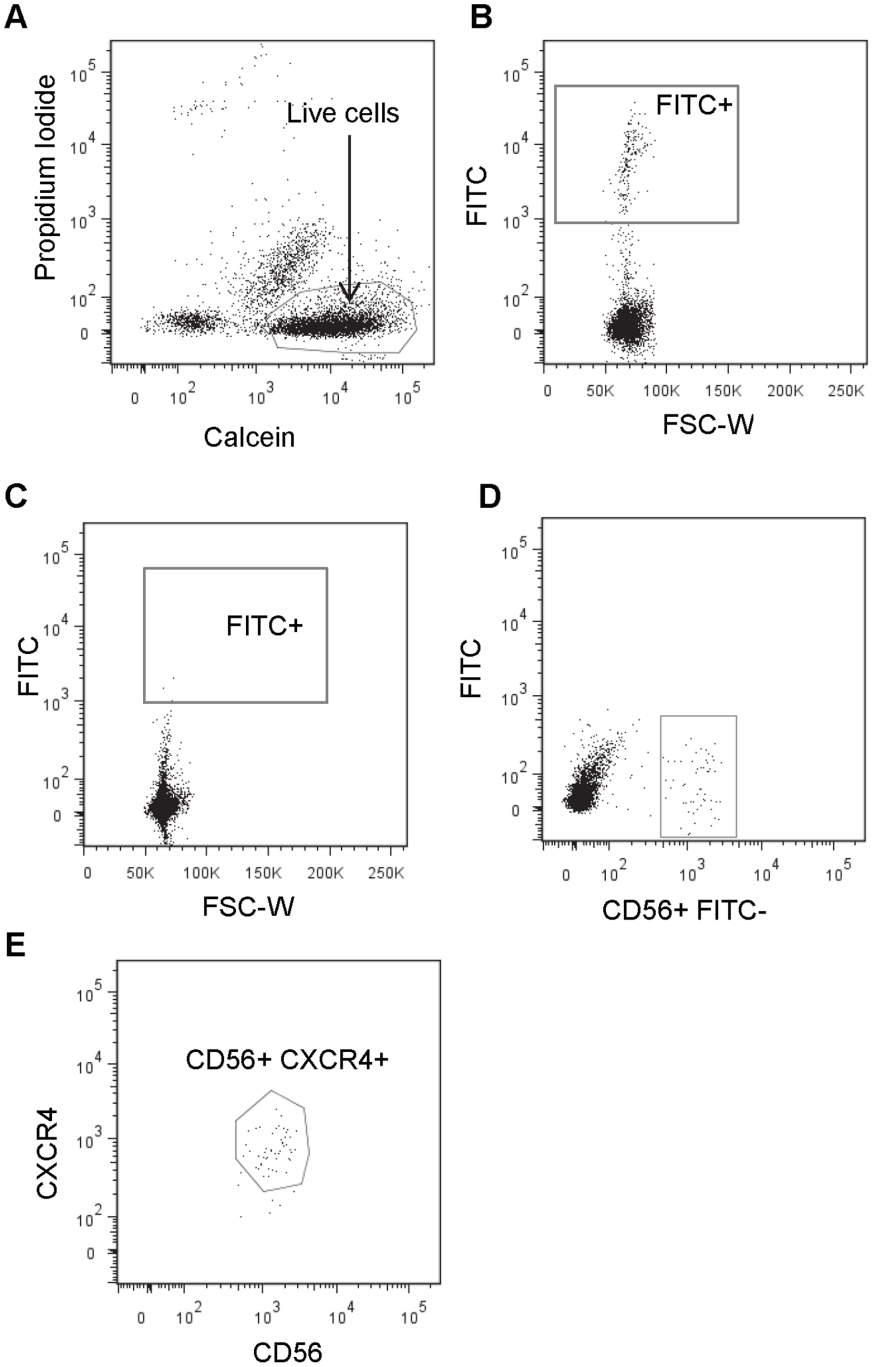
huSMP isolation strategy. A. FACS plot of Calcein blue (live cells) and Propidium Iodide (dead cells) indicating events gated as live cells. B. FACS plot of cells before depletion of the FITC-linked antibody labeled cells. C. FACS plot demonstrating the effectiveness of FITC+ linked antibody labeled cell depletion. D. FACS plot of a sample depleted of FITC+ labeled cells gated on CD56+ cells. E. FACS plot of the CD56+ subset from D showing the CXCR4+, CD56+ subset of cells that were collected for further experimentation.

Previous studies have identified the CD34−/CD56+ population of myogenic cells derived from human skeletal muscle [10]. The method of isolation employed positive and negative selection of cells using antibodies and magnetic separation. Such a technique cannot distinguish between live and dying cells nor can it effectively separate single cells from doublets or larger aggregates. Thus we refined the method based on previous FACS-based isolation strategies developed for mouse muscle [4], [7]. We defined a set of positive and negative markers for selection of cell surface antigens by FACS based on literature reports to define a set of cell surface antigens to interrogate. The negative set included blood markers CD11b and CD45, the endothelial marker CD31, and CD34. Sca1 is widely used as a negative selection marker in the mouse [4], [5], [7], [14]. However, this antigen, encoded by the mouse gene Ly6a, is not encoded by the human genome and thus was not included in our negative selection cocktail. CD34 is known to be expressed by the majority of mouse satellite cells [15]. In contrast, human muscle derived CD34+ cells are myogenic and adipogenic while CD34− cells are myogenic and not adipogenic [10]. CD34 was therefore used as a negative selection marker in our study. Cells expressing these markers (detected by antibodies conjugated to the FITC fluorophore) were depleted by means of magnetic bead separation(Figure 1B,C). CD56 expression has been shown to reliably identify satellite cells in skeletal muscle sections [16], [17], and CXCR4 is expressed by mouse satellite cells [4]. We observed double positive CD56+, CXCR4+ cells that represented approximately 40% of the FITC- population. We observed few CD56+, CXCR4− or CD56−, CXCR4+ cells in the FITC- population. The CD56+, CXCR4+ population was isolated for further experimentation.

The gating strategy used to isolate putative human satellite cells is illustrated in Figure 1. Forward and side scatter gating was performed to select for small, mononuclear cells of low granularity [7]. Following this, the live fraction (calcein blue-positive and propidium iodide-negative) was selected (Figure 1A). Of this live fraction, those cells that were depleted of lineage markers CD11b, CD45, CD31, CD34 but that retained CD56 staining were selected (Figure 1D). The FITC-, CD56+/CXCR4+ cells were then collected into a tube containing an appropriate amount of growth media (Figure 1E). Following a previous convention [13] we term these cells “human skeletal muscle precursors” or huSMPs. These cells accounted for approximately 2% of the total live cell population from undepleted cell mixtures and 10–30% of live cells in the depleted cell mixtures.

### Contribution of huSMPs to *de novo* Muscle Formation in the Mouse

**Figure 2:**
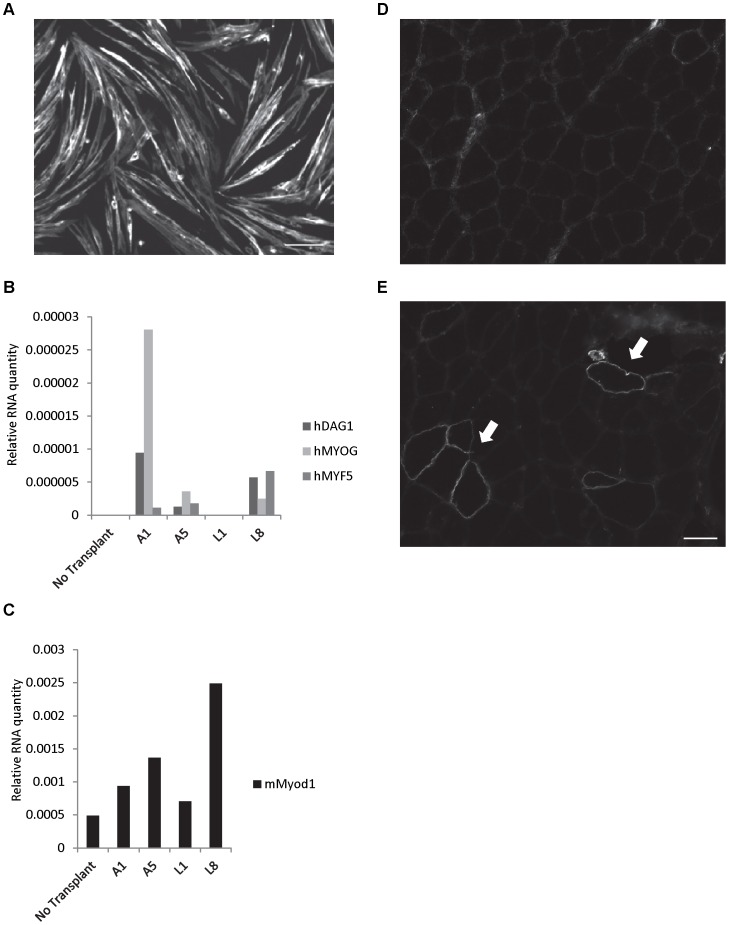
Myogenic activity of transplanted cells. A. Myotube formation by huSMPs after 4 days of differentiation indicated by myosin heavy chain staining. Scale bar = 100 µm. B. qPCR data of RNA extracted from transplanted mouse gastrocnemius muscle. A1, A5, L1, L8 represent individual mice transplanted with two different preparations of huSMPs into the gastrocnemius muscle of one leg. Expression of human myogenic genes is observed in three of the four samples. Relative RNA amount was obtained after normalization to mouse *Gapdh* levels. C. Mouse *MyoD1* expression in all samples indicates that the RNA was of sufficient quality for qPCR analysis in all samples. D. No human DMD+ fibers were observed in control contralateral muscle injected with growth media only. E. Human DMD protein expression in mouse myofibers after transplantation of huSMPs indicates engraftment and differentiation of the cells (arrows). Scale bar = 50 µm.

To validate that the isolation strategy yielded functionally myogenic cells we plated freshly isolated huSMPs and expanded them for 7 days in proliferation conditions and then switched them to myogenic differentiation media for four days. The cells were then fixed and stained for myosin heavy chain. Figure 2A shows a representative field of differentiated myotubes expressing myosin heavy chain confirming the ability of the cells to complete myogenesis *in vitro*. We next performed transplant studies similar to those performed by Pisani et al [10] to determine whether the cells were myogenic *in vivo*. 1×10^3^ freshly-isolated huSMPs were injected into the gastrocnemius muscle of cardiotoxin-damaged *SCID/Beige* mice immediately after isolation. Four weeks after transplantation gastrocnemius samples were collected and RNA was isolated. The RNA was analyzed for the presence of human gene transcripts that would indicate the successful integration of the transplanted cells into the muscle tissue. We identified the expression of human *Dystroglycan* (*DAG1*), *Myogenic Enhancer Factor 5* (*MYF5*), and *Myogenin* (*MYOG*) in three of four transplanted gastrocnemius muscles (Figure 2B). Detection of the mouse *MyoD1* transcripts in all five muscles indicated that the qPCR assay worked as expected (Figure 2C). The presence of human Dystrophin (DMD) protein expressing myofibers in two transplanted muscles (9 and 14 fibers for mice A1 and A5 respectively) was also confirmed by immunofluorescence staining of transplanted muscle using the human DMD specific antibody (Figure 2E). The contralateral control muscle did not contain any such human Dystrophin+ fibres (Figure 2D). Thus, the FACS based isolation strategy isolates cells capable of myogenic differentiation *in vivo* in a manner similar to that described by Pisani et al [9], [10].

### Expression of Myogenic Transcription Factors and Myogenic Potential of huSMPs *in vitro*

**Figure 3:**
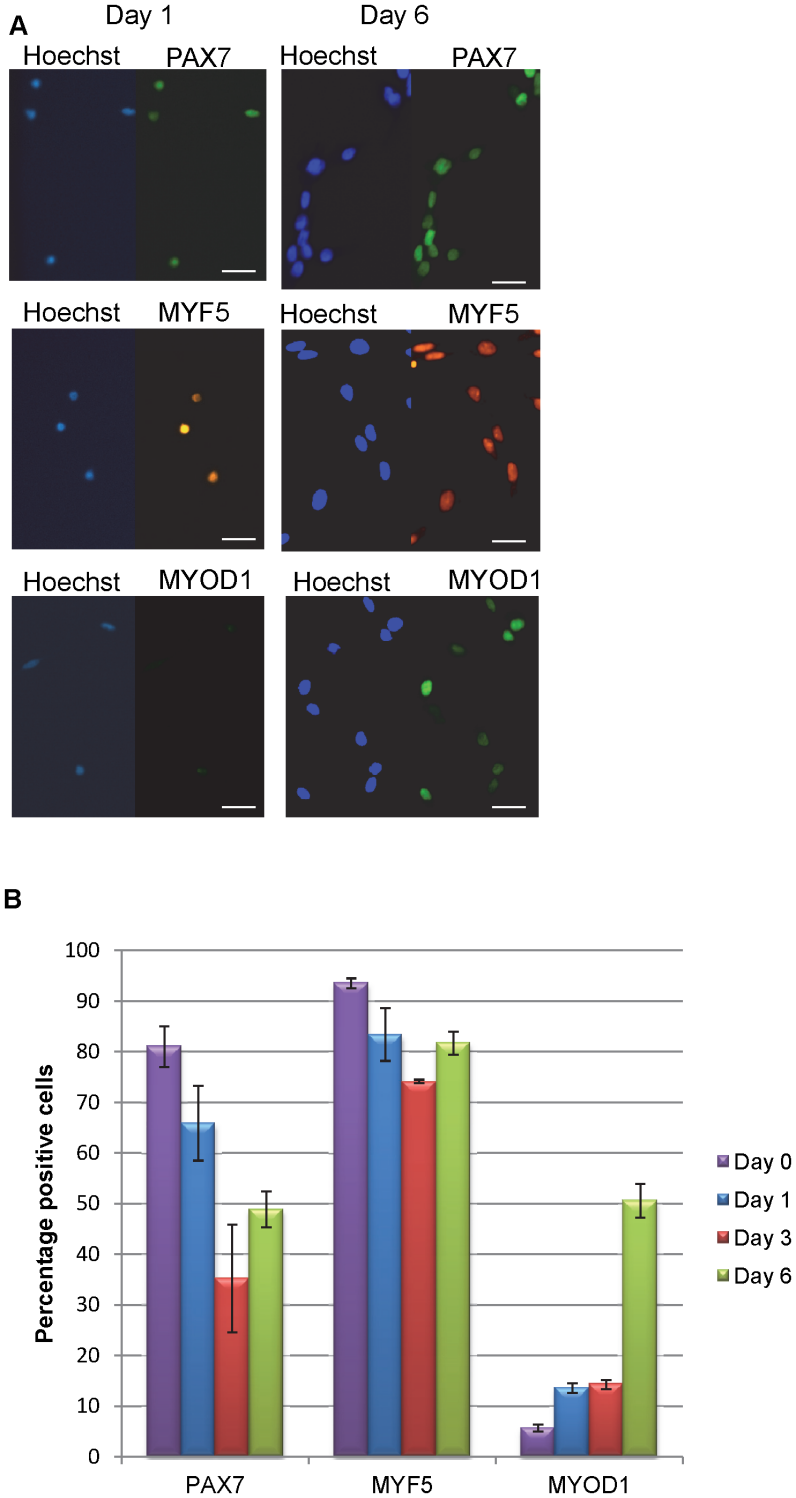
Temporal regulation of Myogenic transcription factor expression. A. Immunofluorescence staining of cultured huSMPs shows increased markers of activation over time (scale bar = 50 µm). B. Quantification of positive staining nuclei as a percentage of total nuclei in the wells.

In mouse satellite cells the expression of myogenic transcription factor proteins is temporally correlated with activation, proliferation, and differentiation. We therefore examined huSMP cultures for the temporal expression pattern of myogenic transcription factors to determine the timing of protein expression (Figure 3A). In freshly isolated huSMPs PAX7 was detected in ∼80% of the cells, MYF5 was detected in ∼90% of the cells, and MYOD1 was detected in ∼5% of the cells (Figure 3B). One day after plating PAX7 could be readily observed in ∼65% of the cells and this declined to ∼35% by day 3 and ∼50% by day 6. Over the same time course MYF5 protein was detected in ∼80% of the cells. Interestingly, MYOD1 protein was found in only a small percentage of cells until day 6 when it was observed in ∼50% of the cells (Figure 3B). This is in contrast to the situation in the mouse where MyoD1 protein expression is considered a hallmark of the activated satellite cell and is observed as early as 24 hours after isolation [18]. After 6 days in culture the cells were transferred to medium containing 2% horse serum without bFGF supplement, and differentiated for 4 days. In addition we tested the myogenic potential of the cells plated as single cells per well and calculated the cloning efficiency. We observed that 34.8+/−11% of the wells (N = 3 donors, 96 wells per donor) contained colonies and all of these demonstrated myosin heavy chain protein expressing myotubes upon differentiation (data not shown).

We also determined whether huSMPs can adopt alternative cell fates. HuSMPs were seeded at 500 cells per well and grown for 7 days. The growth media was then washed out and replaced with myogenic differentiation media (F10 plus 2% heat inactivated horse serum) or commercially available adipogenic or osteogenic media. Differentiation was allowed to proceed for 7 days at which time cells were either fixed and stained with antibodies to myosin heavy chain or harvested for RNA isolation.

**Figure 4:**
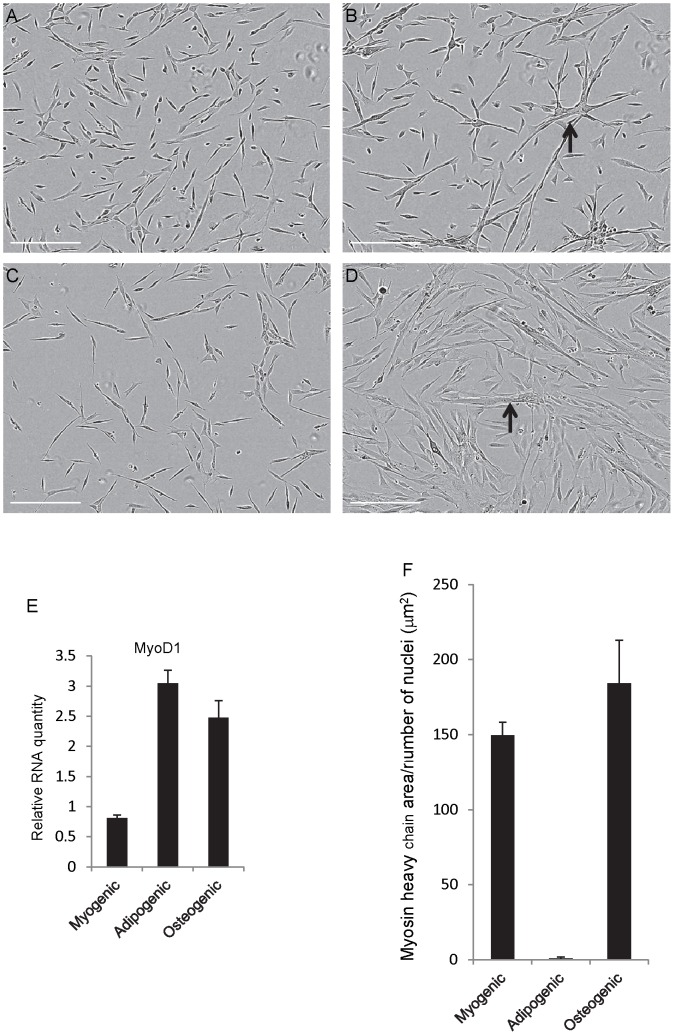
No evidence of lineage changes in huSMP cultures. A. Phase contrast photomicrograph of a proliferating culture of huSMPs just prior to changing to differentiation conditions. B. After 7 days of differentiation in myogenic differentiation media myotubes are readily observed (arrow). C. In adipogenic differentiation media huSMPs do not fuse into myotubes and do not exhibit morphological characteristics of adipocytes such as intracellular lipid droplets. D. In osteogenic media huSMPs still differentiate into myotubes (arrow). E. The expression of *MYOD1* RNA is maintained in all three differentiation conditions. F. Total area of myosin heavy chain staining normalized to the number of nuclei per well is not altered by differentiation in osteogenic media. Adipogenic media appears to block progression to myosin heavy chain expressing myoblast and myotubes. Scale bar = 200 µm.

Inspection of phase contrast micrographs of the cultures revealed the presence of multinucleated myotubes in the myogenic condition and in the osteogenic condition (Figure 4B and D respectively). Interestingly, in the adipogenic conditions the cells appeared to not fuse into myotube like structures (Figure 4C) but appeared to still retain the morphology of the proliferating cultures (Figure 4A). We did not observe intracellular droplets that would indicate differentiation into adipocytes (Figure 4C) nor did we observe staining of the cells with Oil Red O (data not shown).

We next measured myotube formation by myosin heavy chain immunostaining and quantitative microscopy. As expected, cells in myogenic media expressed abundant myosin heavy chain and formed myotubes (Figure 4F). The fusion index for these cultures was 25%+/−1.7%. Interestingly, cells in the adipogenic conditions demonstrated nearly undetectable levels of myosin heavy chain staining indicating slowed differentiation in this media. A fusion index was not calculated as myotubes could not be readily identified due to a lack of myosin heavy chain staining. Osteogenic media supported myogenesis to a similar extent as the myogeinc differentiation media (Figure 4F). The fusion index for the osteogenic condition was 16.2+/1.8%.

We next performed qPCR analysis on the RNA samples and determined the expression levels of the myogenic lineage marker gene *MYOD1* as a measure of myogenic lineage commitment. We also examined the expression of the adipocyte associated transcription factor *Peroxisome Proliferator Activated Receptor Gamma* (*PPARγ)* and the osteo-lineage marker *Integrin Binding Sialoprotein* (*IBSP*) with validated primers. The expression level of the *Beta Actin* RNA was used as an internal reference and for normalization. Neither *PPARγ* nor *IBSP* were detected in any of the three differentiation conditions (data not shown). *MYOD1* was expressed in all three conditions suggesting that a lineage shift away from myogenesis was unlikely to have occurred under adipogenic or osteogenic differentiation conditions (Figure 4E). Based on these results it appears unlikely that lineage switching is occurring under these conditions. However for unknown reasons adipogenic media does appear to slow down or arrest differentiation of the cells at a pre-myosin heavy chain expressing stage.

### Mouse and Human SMPs Display Unique Gene Expression Profiles and Respond Differently to Stimulation by IL1β

**Figure 5:**
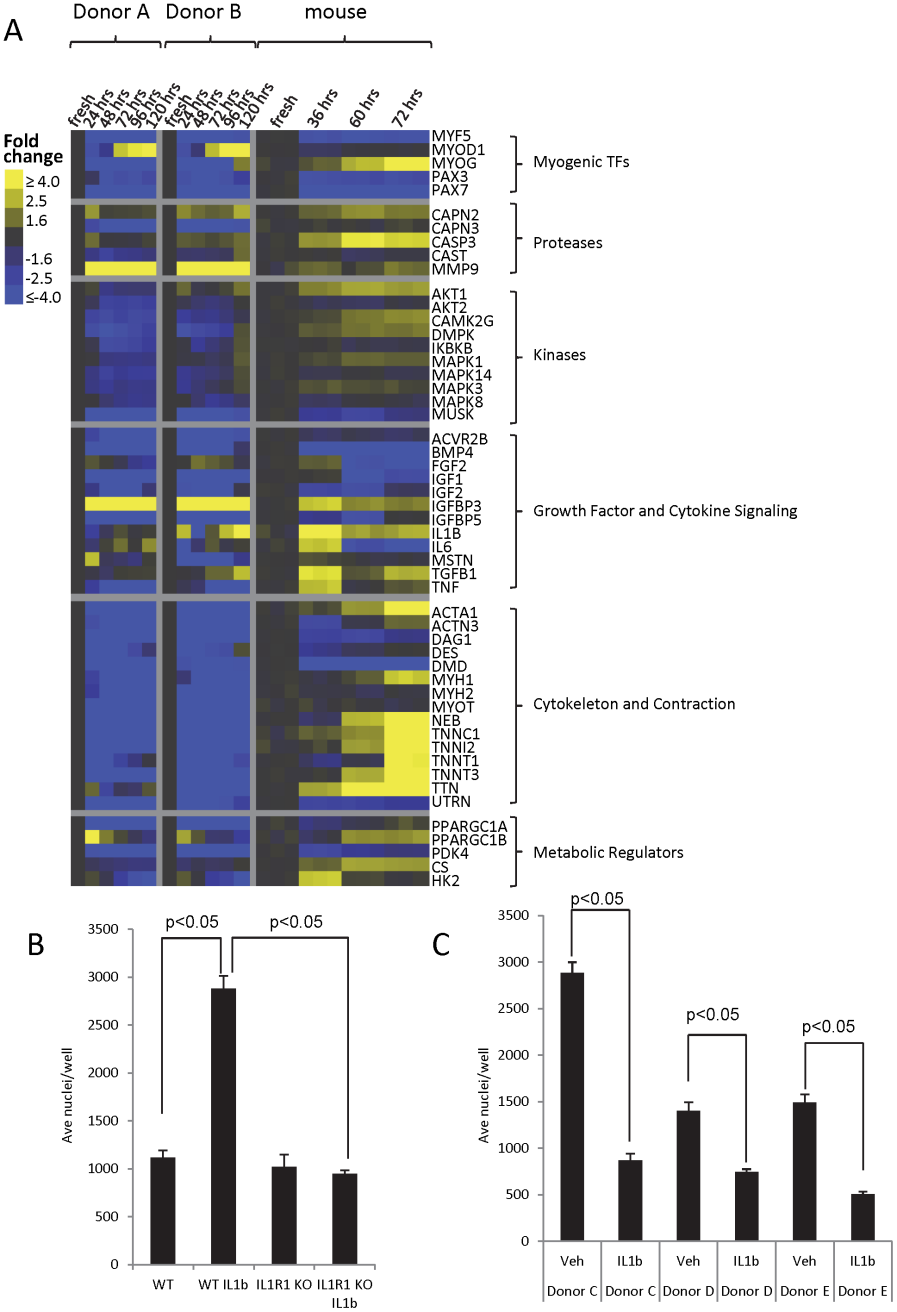
Comparison of mouse and human SMP myogenic programs. A. Gene expression data derived from qPCR assays run with RNA extracted from huSMPs at the indicated time points displayed as a heat map. Numerous differences in the timing of expression of key myogenic genes are readily observed. B. Mouse SMPs cultured in the presence of mouse IL1β (1 ng/ml) have 2.5 times as many SMPs at the end of a five day culture period as compared to cell cultured under standard conditions. Mouse SMPs lacking the IL1β receptor do not respond to the cytokine indicating specificity of the growth response. C. Growth of human SMPs derived from three different donors is reduced by culture in the presence of human IL1β (125 pg/ml).

It is unknown whether human and mouse satellite cell derived muscle precursors use the same gene regulatory pathways during myogenesis. To characterize the huSMP in culture and compare it to the mouse we performed a gene expression profiling experiment using a qPCR array for genes associated with the myogenic process (Figure 5A and Table S1 that contains the normalized gene expression values for all of the genes in Figure 5A). Expression of the RNA for all of the displayed genes was detected in the freshly isolated huSMPs. Strikingly, the expression of most genes was reduced at 24 hours after plating the cells in growth media. This included the classical myogenic transcription factors *MYF5*, *MYOD1*, *PAX3* and *PAX7*. Over the next 4 days in culture *MYOD1* RNA levels increase indicative of the gradual activation of the myogenic gene program. As a comparison we examined the expression of these same genes in mouse SMPs using a published dataset [19]. In the mouse, *MyoD1* RNA is also increased over time after activation. The RNA for *MyoG* is rapidly upregulated in the mouse cells but only begins to increase after 120 hours in huSMPs (donor B). This suggests a more rapid progression to differentiation in the mouse SMP as compared to the huSMP. Consistent with this we observed an increase in expression of genes of the mature contractile apparatus (genes in the category “Cytoskeleton and Contraction”) in mouse SMPs at 60 and72 hours post activation while the huSMPs have not yet activated this aspect of the differentiation program.

The expression of genes associated with metabolic regulation also reveals potentially significant differences between huSMPs and mouse SMPs. *PPARGC1β* is a transcriptional coactivator associated with the regulation of genes involved with oxidative metabolism and angiogenesis [20]. *PPARGc1β* is highly elevated in 24 hr and 48 hr cultures of huSMPs whereas in mouse SMPs it is not induced until 60 hrs after activation, a time point where huSMP expression of *PPARG1β* has waned (Figure 5A, “Metabolic Regulators”). In addition, both citrate synthase (*Cs*) and hexokinase 2 (*Hk2*) are increased in mouse SMPs with activation but are little changed in huSMPs. Taken together this data suggests distinct differences in metabolic activity between activated human and mouse SMPs.

Growth factors and cytokines are known to be key regulatory proteins during myogenesis and in the pathogenesis of muscle disease [21], [22]. We analyzed the expression of eleven genes associated with growth factor and cytokine signaling and identified distinct differences between the human and mouse cell systems. In the mouse, there is a marked induction of *Il1β*, and *Tgfβ1* at 36 hours after activation whereas in the human cells there is no consistent pattern of regulation of these cytokine genes. By contrast, in both the human and mouse cells, *IGFBP3* is markedly induced 24 hours after activation and is maintained at an elevated state compared to the freshly isolated cells.

IL1β has not been previously associated with satellite cell activation so we tested its effects directly on mouse and human SMPs. Mouse SMPs treated with IL1β displayed a dramatic growth phenotype over untreated cells suggesting that IL1β is mitogenic for these cells (Figure 5B). The canonical receptor for IL1β, IL1R1 appears to be responsible for transmitting the signal as mouse SMPs isolated from IL1R1 null muscle tissue do not respond to IL1β Figure 5B). By contrast, human SMPs treated with IL1β displayed a phenotype opposite to that of the mouse cells with reduced numbers of cells after growth in culture (Figure 5C). The number of nuclei in huSMP cultures treated with IL1β was lower than the number of nuclei in untreated cultures. This result was consistent across three different donors, suggesting that is a common feature of huSMPs.

### Effects of the Knockdown of Key Myogenic Genes in huSMPs

**Figure 6:**
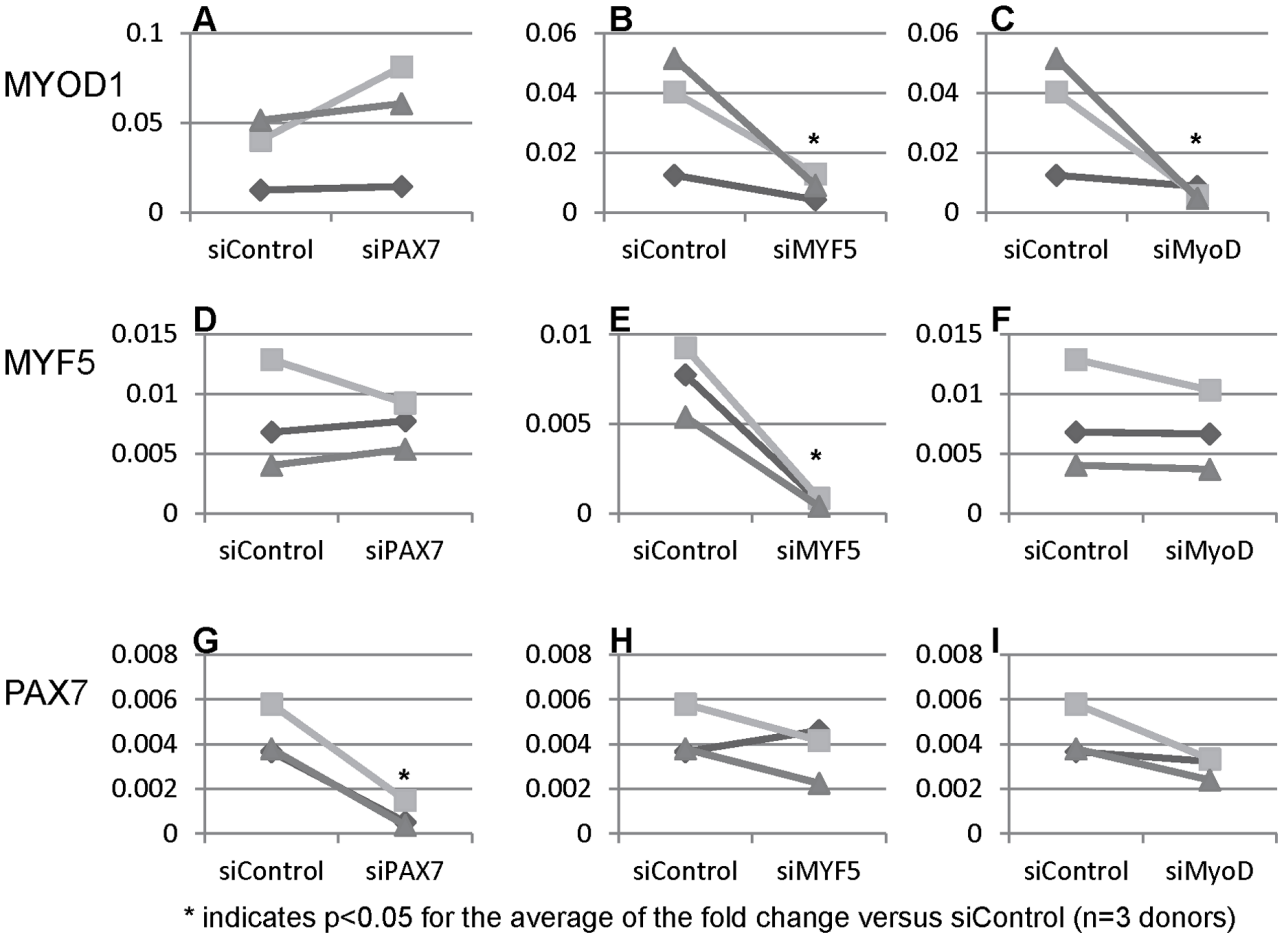
Knockdown of myogenic transcription factors by siRNA reveals regulatory interactions. A. The myogenic transcription factor analyzed by qPCR is listed on the left. siRNA treatments are on the X axis of each graph. Each symbol represents huSMPs derived from a single donor (a total of three donors tested). The matched-pair analysis clearly indicates the knockdown of the cognate transcripts (C, D, and E). B. A significant reduction in *MYF5* RNA as a result of si*MYOD1* treatment is observed. D. *MYF5* expression is unchanged with si*PAX7* a result that is in contrast to that observed in the mouse.

In mouse satellite cells myogenic transcription factors regulate each other’s expression via extensive transcriptional cross talk [23]. To further characterize the myogenic character of the huSMP we used siRNA against *PAX7*, *MYOD1,* or *MYF5* to singly reduce the expression of each transcription factor. We then determined what impact there is on the expression of the RNAs encoding *PAX7*, *MYOD1*, or *MYF5*. In order to assess the consistency of any observed effects we performed the experiment in cells isolated from three separate donors. Transfection of siRNA was performed 1 day after plating the cells and RNA was isolated for analysis 3 days after plating the cells. Each siRNA significantly reduced the level of its cognate target RNA (Figure 6C, E, G). si*PAX7* had no effect on the expression of *MYOD1* or *MYF5* RNA (Figure 6A, D). si*MYF5* dramatically reduced *MYOD1* levels but was without effect on the other myogenic transcription factors (Figure 6B, H). si*MYOD1* was without effect on the other genes (Figure 6 F, I). These data indicate the existence of a positive regulatory interaction wherein *MYF5* directly or indirectly enhances the expression of *MYOD1*.

## Discussion

In the last 10 years it has become possible to isolate populations of satellite cells that express high global levels of *PAX7* and have unambiguously been shown to contribute to muscle regeneration. Although there are minor differences in the protocols developed to isolate these cells, they are all FACS-based and make use of a range of positive and negative selection markers [13], [14], [24]. Research on human satellite cells has lagged behind, and only a fraction of the reported studies have made use of FACS to isolate the cells. The majority of such studies employ methods that include extended periods of culturing [25]–[28], and it has been firmly established that satellite cells rapidly lose quiescence to become committed myogenic cells soon after being exposed to cell culture conditions [7]. The few published studies that have made use of FACS to isolate and examine fresh human satellite cells used fixed cells stained with a PAX7 antibody, and therefore were unable to investigate the behavior of these cells *ex vivo* [29], [30].

Using the isolation procedure described in this study, it was found that approximately 2% of the total live cell population expressed the positive markers and did not express the negative markers. This is in comparison to between ∼4.5–6% for the mouse [13], [31]. The rarity of the cell population is thus within a similar range of frequencies observed for myogenic precursors obtained from mouse muscle. We also observed engraftment and myogenic differentiation of these cells consistent with the results obtained with magnetically isolated CD34−, CD56+ cells [9], [10].

Subsequent analysis of *in vitro* activated and cultured cells demonstrated that huSMPs elaborate a genetic program that is clearly myogenic but with distinct differences from that of the mouse. First, the expression of PAX7 protein remains detectable in up to 50% of the cells even after 6 days in culture a time when few PAX7 expressing cells remain in cultures of mouse SMPs [32], [33]. Similarly, MYOD1 protein is not widely detected in the huSMP cultures until after day 3 of culture, a time when the vast majority of mouse SMPs are expressing the protein [18], [33], [34].

Second, the gene expression array data shows that the temporal expression patterns of many genes are different between mouse and human cells as the cells become activated and begin to differentiate. Although huSMPs began to show an increase in expression of *MYOD1* RNA at an earlier time point, at no point over the course of the 5 days did they show an increase in expression of *MYOG* RNA despite growth of the cultures to densities typically associated with differentiating cultures of mouse SMPs. In contrast, mouse satellite cells show an increase in expression of *MYOG* as early as 60 hours after activation. This indicates that mouse cells display a more precocious ability to differentiate than huSMPs. These results are of potential therapeutic importance because they suggest that it might be possible to expand huSMPs for a period of time before transplanting them into host muscle.

Third, the regulatory relationships between myogenic factors is not completely conserved between mouse and human SMPs. PAX7 is a direct positive regulator of *MYF5* in mouse satellite cells [35], [36] and the PAX7 binding site in the *MYF5* promoter is highly conserved among multiple vertebrate species including *Homo sapiens* [37], [38]. However, we found that knocking down *PAX7* did not have a significant effect on *MYF5* expression in huSMPs. In contrast, siRNA knockdown of *Pax7* in mouse SMPs reduced *Myf5* expression [36]. Thus, despite the conservation of the PAX7 binding site in the *MYF5* promoter, PAX7 does not appear to be an obligate regulator of MYF5 in huSMPs. We also find that knockdown of *MYOD1* in huSMPs has no effect on *MYF5* expression whereas in *MyoD1* null mouse myoblasts Myf5 protein is increased [39]. By contrast, some regulatory relationships are conserved between mouse and human. *MYF5* knockdown resulted in a decreased expression of *MYOD1*, which is in agreement with the fact that mouse *Myf5* is known to be genetically upstream of *Myod1* in the mouse satellite cell, at least during embryonic myogenesis [40]. Together, these findings underscore the existence of fundamental differences between the regulatory circuitry in human and mouse adult myogenesis. Further experimentation will be required to fully elucidate the functional consequences of these differences.

Finally, we demonstrate a previously unappreciated positive effect of IL1β on the growth of mouse SMPs. This result is interesting considering that inflammatory cytokines such as IL1β are generally associated with muscle wasting and may be predicted to inhibit muscle repair [41]. Importantly, we observed that IL1β had a negative effect on huSMP growth, opposite to the positive effect on the growth of mouse SMPs. It is now well appreciated that mouse inflammatory reactions may differ substantially from those in humans and that in general the mouse is a poor predictor of human immune mechanisms [42]. Thus, the opposing responses of mouse and human SMPs to IL1β may represent another facet of the divergence of inflammatory signaling between the species. Such differences should be taken into consideration when examining the role of the immune response to injury of skeletal muscle as they may impact the translation of preclinical experiments to clinical investigations.

## Supporting Information

Table S1 (XLSX)
